# The Photomodification Method Allows for Determining the Composition of the Full and Soft Protein Corona on the Lipid Surface of Composite Nanoparticles

**DOI:** 10.3390/nano14231976

**Published:** 2024-12-09

**Authors:** Anna V. Epanchintseva, Svetlana V. Baranova, Julia E. Poletaeva, Anastasiya V. Tupitsyna, Elena I. Ryabchikova, Ilya S. Dovydenko

**Affiliations:** Institute of Chemical Biology and Fundamental Medicine SB RAS, 630090 Novosibirsk, Russia; annaepanch@niboch.nsc.ru (A.V.E.); swb@niboch.nsc.ru (S.V.B.); poletaeva@niboch.nsc.ru (J.E.P.); tupitsyna@niboch.nsc.ru (A.V.T.)

**Keywords:** multilevel nanoconstruct, hard protein corona, soft protein corona, full protein corona, photofixation, LC MS/MS analysis

## Abstract

A protein corona (PC) is formed and maintained on the surface of any nanoparticle (NP) introduced into biological media. The full PC is formed by a hard and soft corona, and the latter determines the nature of the interaction of NPs with cells and the body’s liquids. Nanomedicines are becoming increasingly important in modern health services, making information about the composition of PCs on the surface of NPs critically important for “managing” the behavior of nano-objects in the body. Currently, only a few studies report on the composition of the complete PC, since the isolation and preservation of the soft corona on the surface of the NP is extremely difficult. Recently, we proposed for the first time a photomodification method to fix PCs on the lipid surface of composite NPs, along with their isolation and purification. In this work, using tandem mass spectrometry, we successively determined the composition of the hard and full corona on the lipid surface of composite NPs, and we also identified the composition of the soft corona. To test the method, we changed the composition of the medium whose proteins formed the soft corona, and we found changes in its composition.

## 1. Introduction

The use of nanoparticles (NPs) for drug delivery allows for the manipulation of such parameters as the solubility of hydrophobic compounds in water, their circulation and half-life time, the conditions and rate of release of the active compound from the dosage form, the possibility of simultaneous delivery of two or more drugs, and immunogenicity. Currently, there are a number of NP-based drugs on the pharmaceutical market [1,2]. The clinical application of nanopreparations reflects the high achievements of nanobiotechnologies, but there is still no complete clarity regarding what happens to nanoparticles when they enter the body. First, this concerns the formation of the so-called biomolecular corona around the NPs in physiological media, the main components of which are protein molecules [3,4,5].

Sorption of proteins from the surrounding physiological environment onto the surface of NPs serves as the main driver of protein corona formation; and it depends on both the physicochemical parameters of the NPs (size, surface curvature, presence and type of charge, surface hydrophobicity) and the properties of the protein molecules, which, in turn, are largely determined by the protein structure [6,7,8].

The duration of corona proteins’ residence on the particle surface varies. Proteins whose residence time significantly exceeds the duration of the experiment form a so-called “hard corona” [9]. Hard corona proteins bind to the NP surface almost irreversibly; hard-corona-bearing NPs can be isolated, and their corona protein composition can be determined [10,11]. Along with the proteins of the hard corona, which are tightly bound to the NPs’ surface, there are proteins with low affinity to the surface, which form an extremely variable layer—the “soft corona”, the composition of which easily changes under the influence of environmental factors [9].

The high instability of the soft corona, determined by its dynamic nature, makes the task of isolating soft-corona-bearing NPs difficult to achieve. Only a few studies have been published on the isolation of full-corona-bearing NPs and determination of the protein composition of both the hard and soft corona [12,13]. For the most part, the soft corona is studied using in situ analytical methods [14].

The formation of a protein corona on the surface of NPs changes their surface and should probably affect their properties, including the behavior of NPs in the body when interacting with target cells [15]. This makes it necessary to take into account the formation of the corona when developing nanopreparations, which starts in vitro. However, the composition of the soft corona is highly variable, and the corona formed on particles in a model system (for example, in serum) will differ greatly from that formed in the body.

Studies of the soft corona in such a complex system as an organism are currently impossible. The transition to in vivo studies requires the development of effective approaches to determining the composition of the soft corona in model systems. This could not only be a step towards moving research to the organism level but also allow us to get closer to understanding the mechanisms underlying the natural phenomenon of the protein corona.

This work is devoted to studying the possibility of determining the composition of the soft corona formed on the lipid surface of multilevel nanoconstructs (MLNCs), using the photomodification method.

**Figure 1:**
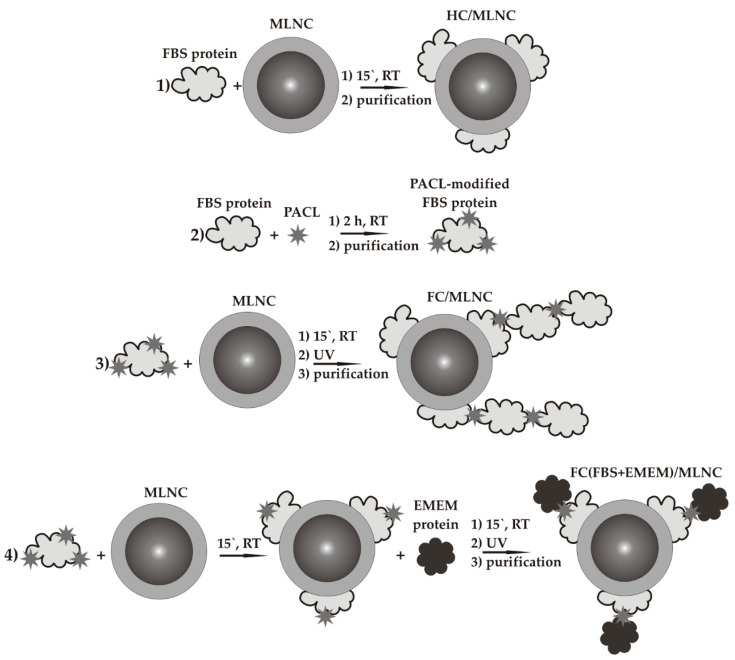
The scheme of the experiment: Stage 1—Obtaining MLNCs bearing a hard protein corona (HC/MLNC) on their surface. Stage 2—Modification of fetal bovine serum (FBS) proteins with PACL. Stage 3—Obtaining MLNCs bearing a fixed full protein corona on their surface consisting only of FBS proteins (FC/MLNCs). Stage 4—Obtaining MLNCs bearing a fixed full protein corona on their surface, consisting of proteins from two sources (FC(FBS+EMEM)/MLNCs).

The experimental scheme is shown in Figure 1. First, we incubated MLNCs with fetal bovine serum (FBS) to obtain hard-corona-bearing particles (Figure 1, stage 1). Previously, we proposed a method for fixing full corona proteins using a photoactivated cross-linking agent (PACL) [16]. The essence of this method is that the proteins of the biological fluid (serum) are subject to modification with the PACL (Figure 1, stage 2).

The PACL-modified FBS proteins then interact with the NP surface to form a corona. Irradiation with ultraviolet light of a specific wavelength allows for covalent cross-linking of spatially close proteins—in this case, the corona proteins (Figure 1, stage 3). The difference between our proposed method and the method based on click chemistry [12] is that the modification that we used will also bind soft corona proteins, which have no contact with hard corona proteins. At the same time, the method using click chemistry only allows for the binding of the soft corona proteins that were sorbed onto the modified hard corona proteins. The modification type that we used allows for collecting a larger volume of information about the composition of the full corona.

We chose MLNCs as model particles on which the protein corona is formed, since their surface is a lipid bilayer and their properties make it easy and safe to isolate and purify corona-bearing particles from unbound proteins [16]. Isolation of the resulting corona-bearing NPs without corona loss is a critical step in the research, and in the case of MLNCs, such recovery can be achieved through the presence of a heavy core consisting of AuNPs.

In this work, we determined the composition of the full corona, which was then compared with the composition of the hard corona. The differences in the composition of the coronas provided us with information about the composition of the soft corona. When changing the biological environment, changes in the composition of the soft corona were recorded, which indicates the efficiency of the photomodification method and its applicability for the isolation of corona-bearing particles.

## 2. Materials and Methods

### 2.1. Preparation of the MLNCs

The MLNCs were assembled step by step: preparation of (i) AuNPs, (ii) lipid films and cores (AuNPs coated with siRNA), and (iii) encapsulation of the cores in a lipid envelope. The HAuCl_4_ (Aurat PAO, Moscow, Russia) was used for the preparation of AuNPs using the citrate reduction method [17]. To obtain MLNCs, AuNPs with d = 12.7 ± 2.0 nm (TEM data) and d = 17 ± 2 nm (DLS data) were used, the ζ-potential was −33.6 ± 2.0 mV.

The gold cores were obtained as described in [18]: 3.6 nM citrate-stabilized AuNPs were mixed with 0.7 μM siRNA, 5.6 mM NaCl (Honeywell, Seelze, Germany), and 0.1 mM MgSO_4_ (Honeywell, Seelze, Germany) and kept for 22 h at 25 °C. The resulting samples were centrifuged at 16,100× *g* for 40 min at 25 °C, and the supernatants were removed.

Lipid films were produced as described in [19]. Solutions of 1 mM egg phosphatidylcholine (Avanti, Alabaster, AL, USA) and DOPE (Avanti, Alabaster, AL, USA) in a CHCl_3_/CH_3_OH mixture (Reachem, Moscow, Russia) (1:1) (90 μL), and 1 mM DOME2 (2-[[4-dodecylamino-6-oleylamino-1,3,5-triazine-2yl]-(2-hydroxyethyl)amino]ethanol) in CHCl_3_ (10 μL), were mixed and diluted in 1 mL of CHCl_3_ in a 10 mL round-bottomed flask. The solvent was vaporized at 12 mmHg without heating. To eliminate any traces of organic solvent, the obtained lipid film was additionally dried under vacuum in a desiccator, and then the film-containing vial was kept at −18 °C for 16 h.

An argon atmosphere was used to perform all of the described manipulations with the lipids and lipid films.

The complete assembly of the MLNCs was as presented in [19]. In brief, the sample of core NPs was diluted in water to a volume of 900 μL, and then added to 31 μL of 10 mM NaH_2_PO_4_ (Reatex, Moscow, Russia) at pH 4.5. The obtained suspension was applied to the lipid film and sonicated for 15 min (25 °C, 90 W). Then, 69 μL of 0.01 M Na_2_HPO_4_ (Reatex, Moscow, Russia) was added to the suspension, followed by 10 μL of 1 mM stearic-acid-conjugated peptide, Str-(RL)_4_G-NH_2_ (Alambion, Voronezh, Russia); the resulting suspension was sonicated for 5 min (25 °C, 90 W).

Purification of the MLNCs from empty lipid particles and free cores was performed by centrifugation of the suspension on a cushion of 75% glycerol in 1 mM phosphate buffer (PB) at 2000× *g* and 25 °C for 15 min. The fraction of the MLNCs was collected, diluted with 1 mM PBS, and then concentrated by centrifugation at 3000× *g* and 25 °C for 10 min. The purified MLNCs had good colloidal stability, and the average ζ-potential for several MLNC samples was −35.2 ± 0.2 mV.

The MLNC samples were mixtures of NPs (up to 100 nm in size) and submicron particles (up to 200 nm) at varying ratios. For brevity, we will refer to the MLNCs as “particles”.

### 2.2. Modification of FBS with PACL

The photoactivated cross-linking agent (PACL) (4-azido-N-[3-[3-(2,5-dioxopyrrol-1-l)propanoylamino]propyl]-2-nitrobenzamide) was prepared in the Organic Synthesis Laboratory (ICBFM SB RAS, Novosibirsk, Russia). Fetal bovine serum (FBS) A3160801, produced by Thermo Fisher Scientific (Waltham, MA, USA), was used in all experiments.

All experiments with the PACL were carried out in a dark box under red light, since it is light sensitive. To modify the FBS, 12 µL of 0.5 M PACL in DMSO was mixed with 1 mL of 50% FBS in 1 mM PB and incubated with constant stirring (400 rpm, 20 °C) for two hours. Surplus PACL was removed using Bio-Spin 6 columns (Bio-Rad, Hercules, CA, USA), as recommended by the manufacturer.

### 2.3. Preparation of Hard Protein Corona-Bearing MLNCs

A 2 mL suspension of MLNCs (40 pmol, determined by AuNPs’ optical density) and 0.5 mL of a 50% solution of (i) FBS or (ii) PACL-modified FBS in 1 mM PB were mixed and incubated for 15 min at 25 °C. The resulting particles were either purified (Section 2.5) and used as hard-corona-bearing MLNCs (control) or used to prepare full-corona-bearing particles.

### 2.4. Preparation of Full Protein Corona-Bearing MLNCs

The hard-corona-bearing particles (Section 2.3) obtained using PACL-modified FBS were irradiated with UV light (310 nm wavelength, 8.2 mW/cm^2^ power) for 1 min to bind the PACL-modified proteins to the MLNC surface. Thus, we obtained full-corona-bearing particles (FC/MLNC), and the full corona was composed only of FBS proteins.

Another kind of full-corona-bearing MLNC was prepared using EMEM culture medium (Sigma–Aldrich, St. Louis, MO, USA) produced from the human HEK293 cell line. The cells grew for 48 h at 37 °C and 5% CO_2_ in EMEM with 1 g/L glucose, in the presence of 10% FBS, 100 U/mL penicillin, and 100 μg/mL streptomycin (Thermo Fisher Scientific, Waltham, MA, USA). After cultivation, the medium was collected and cleared of cell debris by centrifugation (12,000× *g*, 10 min) at room temperature.

A suspension of hard-corona-bearing MLNCs (Section 2.3), obtained using PACL-modified FBS, was diluted with 2.5 mL of EMEM medium and kept for 15 min at 25 °C. In order to bind EMEM and PACL-modified FBS proteins to the particle surface, the MLNC suspension was UV-irradiated as described above. Thus, we obtained particles with a full corona (FC(FBS+EMEM)/MLNC), and in this case the full corona consisted of FBS and EMEM proteins.

### 2.5. Isolation of Corona-Bearing MLNCs

The resulting corona-bearing MLNCs were cleared of unbound proteins by centrifugation in 75% glycerol containing 1 mM PB at 2000× *g* and 25 °C for 15 min, as described in [18]. The colored fractions containing the modified MLNCs were collected and subjected to subsequent purification, concentration, and analysis. 

### 2.6. Washing and Concentration of Isolated MNLCs

To wash FC/MLNC and FC(FBS+EMEM)/MLNC from free proteins and increase the concentration of corona-bearing MLNCs, several identical MLNC suspensions were combined in a 15 mL tube. This suspension was diluted 3.75-fold with 1 mM PB and centrifuged (3000× *g*, 25 °C) for 10 min, and the supernatant was discarded. The MLNC-containing pellet was washed again with 1.5 mL of 1 mM PB and centrifuged (3000× *g*, 25 °C) for 10 min. The supernatant was discarded, and the procedure was repeated twice. Four washes of the MLNC pellet ensured that the preparation was pure enough for analysis.

### 2.7. Digestion with Trypsin

Trypsin digestion was performed according to the manufacturer’s recommendations using ProteaseMAX™ Surfactant Trypsin Enhancer (Promega, Madison, WI, USA), with modifications. We used 5–10 µL of corona-bearing MLNCs for trypsinization: (i) HC/MLNC, (ii) FC/MLNC, and (iii) (FC(FBS+EMEM)/MLNC. Each corona-bearing MLNC sample was added to 2 μL of 1% surfactant (Promega, Madison, WI, USA) in 50 mM ammonium bicarbonate buffer (pH 7.8), and then incubated for 1 min at 25 °C.

Naked MLNCs (lost corona proteins) were removed from each sample by centrifugation (12,000× *g*, 30 min) at 25 °C. The supernatant was placed in a clean tube and 50 mM bicarbonate buffer (pH 7.8) was added to 87.74 μL, and then 1 μL of 0.5 M DTT (Molekula GmbH, Munich, Germany) was added. The mixture was incubated for 20 min at 56 °C.

After restoration of S-S bonds, each sample was mixed with 2.7 μL of 0.55 M iodoacetamide (Shanghai Macklin Biochemical Technology, Shanghai, China) and incubated in the dark for 20 min at 25 °C. Digestion was stopped by adding a mixture of 7.56 μL (1.8 μg) of trypsin (Promega, Madison, WI, USA) and 1 μL of 1% surfactant. Trypsinolysis was carried out for 3 h at 37 °C with shaking at 800 rpm and was stopped by adding trifluoroacetic acid (Sigma-Aldrich, St. Louis, MO, USA) to a final concentration of 0.5%, followed by incubation for 1 min on ice. The peptide mixture was centrifuged (12,000× *g*, 10 min) at 25 °C and placed in vials for subsequent analysis.

### 2.8. LC MS/MS Analysis

The LC MS/MS analysis was carried out at the Core Facility of Mass-Spectrometric Analysis of ICBFM SB RAS. Trypsin-digested peptides were analyzed on an Orbitrap Q Ex-298 high-resolution active mass spectrometer (Thermo Fisher Scientific, Waltham, MA, USA) in the range of 200–1600 Da, with separation on a Poroshell 120 EC-C18 column (Agilent Tech, Waldbronn, Germany). Peptides were washed with 2% phase B (0.1% formic acid and 100% acetonitrile) at a flow rate of 250 mL/min for 3 min, and then eluted with a gradient of B (2% to 40%) over 8 min and a gradient of B (40% to 95%) over 25 min. The sputter voltage was set at 4.2 kV, and the normalized collision energy was set at 30% for MS/MS. Data-dependent ion selection was carried out using the 10 most abundant ions from the full MS scan for MS/MS analysis.

Identification of peptides and proteins was performed using Proteome Discoverer software (version 3.1, Thermo Fischer Scientific, Waltham, MA, USA), using the SEQUEST algorithm. The search was performed in the *Bos Taurus* database or in both the *Bos Taurus* and *Homo sapiens* databases, which were obtained from the UniProt and the reviewed SwissProt database (https://www.uniprot.org accessed on 25 April 2024).

The following settings were used: MS error tolerance 10 ppm, MS/MS error tolerance 0.02 Da, the protease—trypsin, variable modification—oxidation (M), and fixed modification—carbamidomethyl (C), with a peptide confidence level of medium and an average level of peptide reliability.

At least three samples for each corona type were analyzed. Preparation of protein corona-bearing particles and corona composition analysis for each sample were performed independently.

### 2.9. Transmission Electron Microscopy

The materials for TEM were supplied by EMS (Houston, TX, USA).

All samples for TEM analysis were prepared in the same way: 10 μL of the particle suspension was applied to a 200-mesh copper grid coated with a carbon-stabilized formvar film for 1 min. The liquid was then removed with filter paper and the grid (film side down) was placed on a drop of 0.5% aqueous uranyl acetate for 5–10 s for contrast. The solution was collected with filter paper, and the grids were air-dried.

Negatively stained samples were examined using a Jem1400 transmission electron microscope (JEOL, Tokyo, Japan), and images were collected using a Veleta digital camera (EM SIS, Münster, Germany).

## 3. Results and Discussion

The soft protein corona formed on the surface of NPs and submicron particles in biological environments is extremely unstable [9]. Soft corona proteins are in dynamic equilibrium with the environment, which determines the high variability of the corona composition and complicates the isolation and study of the corona in its natural state [3,4,5,6]. Determining the protein composition of the soft corona is a particularly challenging task. An effective approach is to fix the full corona, which includes the soft corona, on the surface of the particles and then determine the protein composition of the full corona and its components—the hard and soft corona.

**Figure 2:**
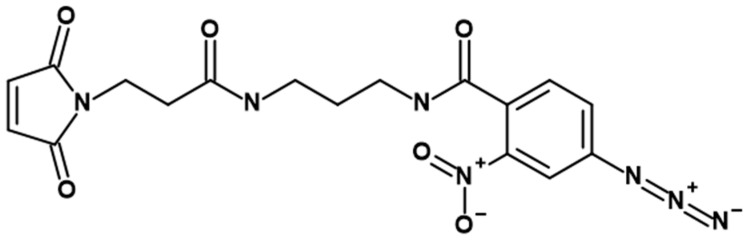
Chemical structure of photoactivated cross-linking agent (PACL).

We proposed using a photoactivated cross-linking agent (PACL) (Figure 2) to fix the components of the protein corona on the lipid surface of MLNCs [16].

The essence of the method is to fix absolutely all proteins on the particle surface, i.e., proteins of the hard and soft coronas. The PACL structure has two functional groups: The first is a maleimide residue, which is able to modify proteins at thiol or amino groups. The second is a photoactivatable group (nitroaryl azide residue) that, when exposed to light of a certain wavelength, forms a covalent bond with spatially proximate PACL-modified proteins localized in the MLNCs’ vicinity. The analysis of the properties and justification for the choice of PACL were as presented in [16].

In this way, it is possible to fix the complete protein complex of the full protein corona on the particle surface. Using MLNCs as a model particle allows for the easy removal of unbound proteins and isolation of pure MLNCs bearing a full protein corona [16].

In this work, we tried to test the possibility of using the method of photofixation of the full corona to determine the composition of the soft corona, which, together with the hard corona, forms the complete protein corona. The problem of identification of the soft corona’s composition can be solved if the protein composition of the full and the hard coronas is known. The hard corona’s composition can be easily determined, while the composition of the full corona can be determined after its fixation on the surface of the particle. This is the approach that we implemented in this work.

To determine the composition of the soft corona, two parallel experiments must be conducted: (i) isolate particles carrying the hard corona and determine its composition; (ii) isolate particles bearing the photofixed full corona and determine its protein composition. Then, by comparing the components of the hard and full coronas, the composition of the soft corona can be determined.

**Figure 3:**
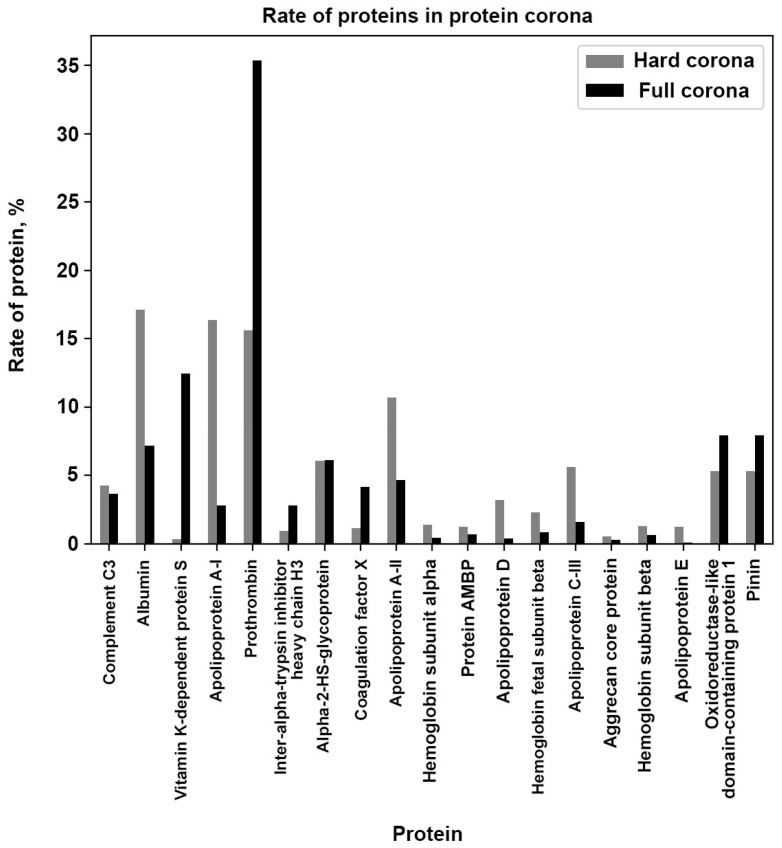
Ratio of protein rates in the composition of hard (grey bars) and full coronas (black bars).

Proteins of the hard corona bind to the surface of particles irreversibly, which allows for easy isolation of hard-corona-carrying particles. Previously, we demonstrated the isolation of purified HC/MLNC samples and determined the hard corona composition [11]. The developed technology was applied in this study to the photomodified MLNCs bearing the full corona. Comparative analysis showed that the diversity of full corona proteins does not significantly differ from the diversity of hard corona proteins (Figure 3; Table 1 and Table S1 (HC/MLNC data); Table S2 (FC/MLNC data)).

The difference between the full and hard coronas is the abundance of particular proteins. As noted above, determining the composition of the soft corona is a complicated task, and studies devoted to solving this problem are extremely rare. In these few studies, researchers also note that the soft corona’s composition is determined by the composition of the hard corona [12,13].

Conceptually, the work of H. Mohammad Beigi and co-authors [12] is closest to our work; both studies propose methods for fixing the soft corona. To study the composition of the soft corona, the authors applied fixation using click chemistry methods. For this purpose, the proteins of the hard corona on the surface of silica and polystyrene NPs were modified using a modifier with an azide group. Separately, the serum proteins were modified with a modifier with a DABCO group. By mixing NPs bearing a hard corona consisting of modified proteins with modified serum proteins, it was possible to fix soft corona proteins on the surface of the NPs bearing the hard corona.

Using silica and polystyrene NPs as an example, it was shown that, in an FBS solution, the soft corona is formed from the same set of proteins as the hard corona. Despite the elegance of the method proposed by H. Mohammad-Beige and co-authors, it is worth noting that it is possible to bind only those soft corona proteins that have interacted with modified HC proteins. This means that if the soft corona contains proteins associated with neighboring soft corona proteins or with the surface of the NPs, they will be irreversibly lost during washing and, accordingly, the information about the composition of the soft corona will be incomplete. The use of PACL ensures the fixation of all spatially proximate proteins in vicinity to the MLNC surface, which provides more complete information on the composition of the soft corona.

It is interesting that, with both experimental approaches, the composition of the full corona was identical to the composition of the hard corona, with the only difference being that the ratio of proteins changed. The conclusion suggests itself that the hard corona contains only those proteins or protein complexes that are capable of binding to the particle surface. At the same time, there are proteins that are capable of binding both to each other and to the surface of the particle. In this case, these proteins will be found in the composition of both coronas.

Comparing the proportions of proteins in the hard corona and full corona, we can see a significant increase in the proportion of such proteins as vitamin K-dependent protein S, prothrombin, coagulation factor X, and inter-alpha-trypsin inhibitor heavy chain H3 in the full corona (Figure 3, Table 1). Interestingly, the proportion of vitamin K-dependent protein S in the full corona increased by 35.24 times compared to its proportion in the hard corona. The increase in the proportions of prothrombin, coagulation factor X, and inter-alpha-trypsin inhibitor heavy chain H3 in the full corona composition, relative to their representation in the hard corona, was 2.26, 3.59, and 3.04 times, respectively. At the same time, the proportions of proteins that were dominant in the composition of the hard corona (albumin, apolipoprotein A-I, apolipoprotein A-II, apolipoprotein C-III, apolipoprotein D, apolipoprotein E) decreased in the full corona. This leads us to the hypothesis that the soft corona is largely composed of vitamin K-dependent protein S, prothrombin, coagulation factor X, and inter-alpha-trypsin inhibitor heavy chain H3.

**Figure 4:**
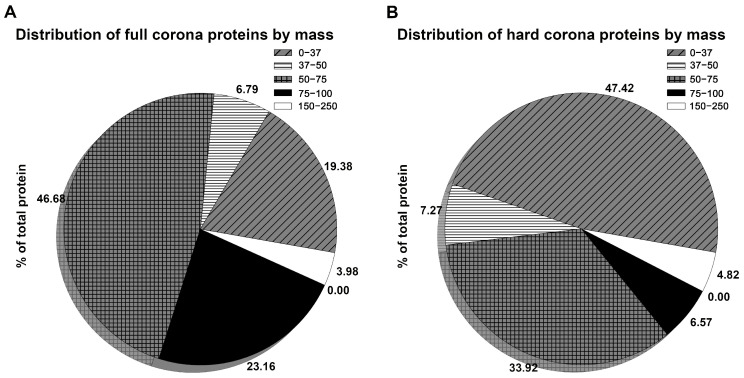
Distribution of proteins by their molecular weight for (**A**) the full corona and (**B**) the hard corona. Values are given in kDa.

Next, we analyzed the distribution of proteins by their molecular weight in the full corona (Figure 4A) and the hard corona (Figure 4B).

The majority of the hard corona is made up of proteins with a mass of up to 37 kDa, as well as proteins whose molecular masses are in the range from 50 to 75 kDa (Figure 4B). In the full corona, the distribution of proteins by their molecular mass changes, so the proportion of “small” proteins, weighing up to 37 kDa, decreases more than twofold. At the same time, the proportion of proteins with a mass in the range from 75 to 100 kDa increases by 3.5 times.

**Figure 5:**
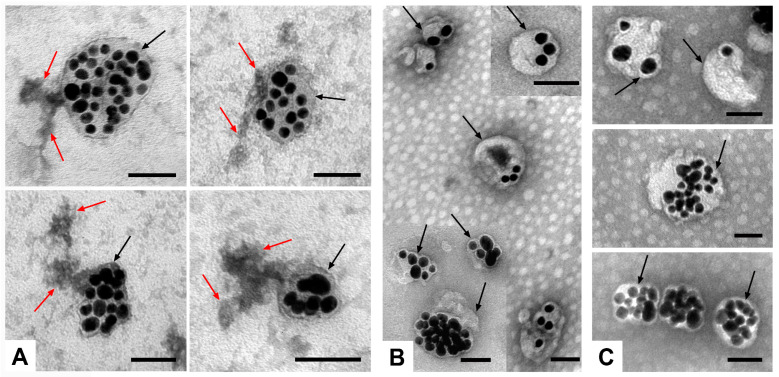
Representative images of MLNCs: (**A**) MLNCs bearing full corona (shown with red arrows); (**B**) initial MLNCs; (**C**) MLNCs bearing hard corona (not visible). Surface of MLNCs is shown with black arrows. TEM, negative staining. Length of scale bars corresponds to 50 nm.

The formation of a full corona was detected on the surface of the MLNCs by TEM (Figure 5A). Large accumulations of a substance with different electron density were observed, which did not have clear outlines. Electron-dense “isthmuses” connecting the corona with the surface of the MLNCs were often visible. In contrast, the initial particles had a smooth surface, not associated with any substance (Figure 5B). MLNCs bearing a hard corona were similar to the initial particles, where the corona was not visible (Figure 5C).

We previously suggested that the differences in corona visualization are due to the small size of the proteins in the hard corona [11]. The results of this study support this suggestion: the full corona had a significantly increased proportion of “large” proteins with masses in the range of 50 to 100 kDa, allowing us to see the full corona. Table 2 shows a detailed distribution of proteins by mass in the hard and full coronas.

These data show that the proteins vitamin K-dependent protein S, prothrombin, coagulation factor X, and inter-alpha-trypsin inhibitor heavy chain H3, the proportion of which in the corona composition increased during the transition from a hard corona to a full one (Table 2), have molecular masses in the range of 50–100 kDa. This once again confirms that the soft corona is formed mainly by the proteins vitamin K-dependent protein S, prothrombin, coagulation factor X, inter-alpha-trypsin inhibitor heavy chain H3.

To finally confirm this, we conducted an additional experiment. In the first stage of the experiment, we formed a hard corona on the MLNC surface in an FBS solution. The resulting hard-corona-bearing particles were mixed with EMEM culture medium collected after 48 h culturing of HEK-293 cells. Since hard corona proteins bind irreversibly to the MLNC surface, we thereby varied the composition of the soft corona. Accordingly, with the help of mass analysis, we should see changes in the composition of the full corona.

**Figure 6:**
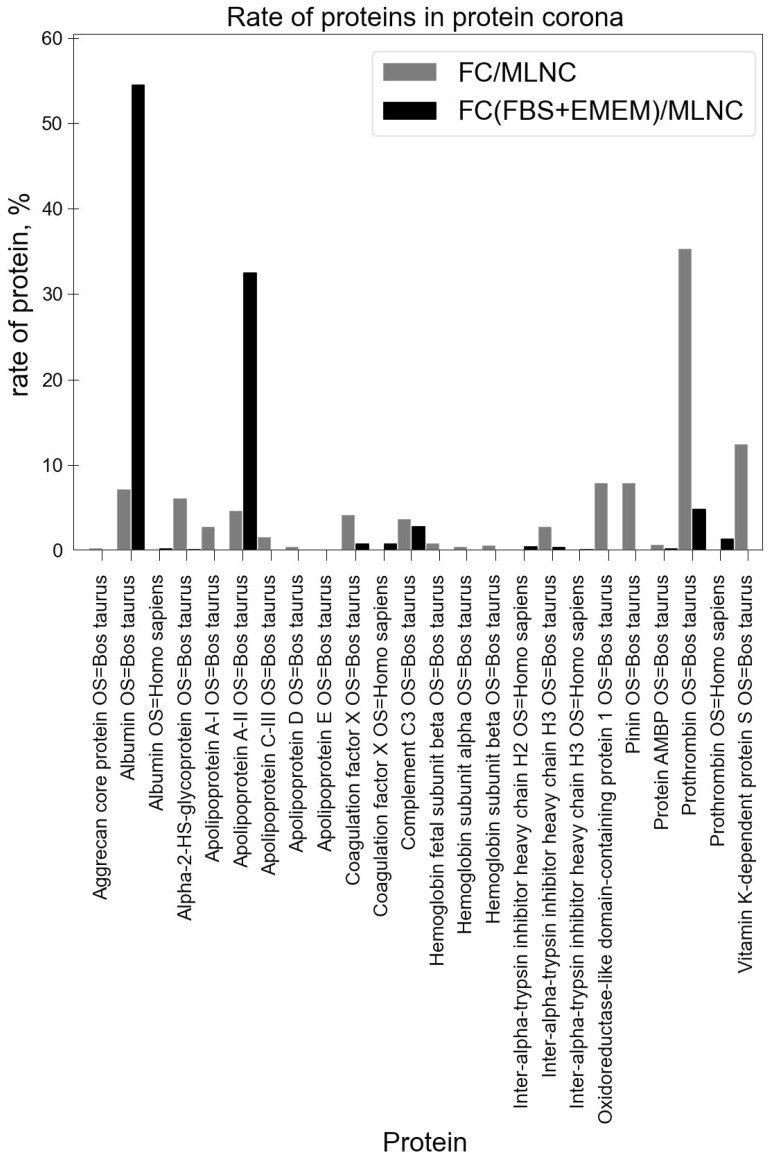
Comparison of protein composition of full coronas for FC/MLNCs and FC(FBS+EMEM)/MLNCs.

A comparison of the compositions of the full protein corona obtained using FBS solution alone and the protein corona obtained using FBS and EMEM solutions is shown in Figure 6 and Table S3 (FC(FBS+EMEM)/MLNC data).

As expected, the composition of the full coronas differed greatly; the variation in the content of the external environment led to a change in the composition of the soft corona. The photomodification method that we proposed allowed for the detection of these changes. In the composition of the full corona obtained using FBS and EMEM culture medium, we observed a decrease in the proportions of vitamin K-dependent protein S, prothrombin, coagulation factor X, and inter-alpha-trypsin inhibitor heavy chain H3, fully confirming our hypothesis that these are the main proteins of the soft corona.

## 4. Conclusions

The method that we developed for fixing the full corona using photomodification provides the preservation of the corona proteins on the lipid surface of MLNCs in the process of isolation and washing from unbound proteins in the biological environment. The implementation of this method in the presence of the photoactivatable cross-linking agent (PACL) (4-azido-N-[3-[3-(2,5-dioxopyrrole-1-l)propanoylamino]propyl]-2-nitrobenzamide), or without it, allows for obtaining particles with a full or hard corona. Subsequently, by comparing the results, it is possible to determine the composition of the soft corona and analyze changes in the full corona.

In this work, we have once again confirmed the results obtained previously by other researchers [12,13]: the hard corona determines the composition of the full corona, and the difference lies in the redistribution of protein portions relative to each other. A comparison of the composition of the hard and full coronas formed on the lipid surface of the MLNCs showed that the main proteins of the soft corona are vitamin K-dependent protein S, prothrombin, coagulation factor X, and inter-alpha-trypsin inhibitor heavy chain H3. When we changed the composition of the biological environment surrounding the particles, the composition of the full corona changed too, and the proportions of these proteins decreased. In this regard, it can be said with confidence that it is these proteins that make up the main part of the soft corona.

The photomodification method allows for fixing the full corona on the lipid surface of composite particles and influencing the composition of the soft corona by controlled changes in the contents of the surrounding environment. As we can see, against the background of the growth in the share of the abovementioned proteins in the composition of the full corona, the share of the main proteins in the hard corona (albumin and apolipoproteins) in the composition of the full corona falls. This suggests that albumin and apolipoproteins are mainly hard corona proteins and are bound directly to the lipid surface of the MLNCs. Thus, “targeting” proteins, such as apolipoprotein E, will be present on the surface of the particles permanently.

## Figures and Tables

**Table 1:** Proportions of proteins in the composition of the hard and complete coronas.

#	Protein	Rate of Proteins in Hard Corona	Rate of Proteins in Full Corona	Relative Magnitude of Change
1	Complement C3	4.27	3.67	0.86
2	Albumin	17.14	7.15	0.42
3	Vitamin K-dependent protein S	0.35	12.44	35.24
4	Apolipoprotein A-I	16.39	2.80	0.17
5	Prothrombin	15.62	35.37	2.26
6	Inter-alpha-trypsin inhibitor heavy chain H3	0.93	2.82	3.04
7	Alpha-2-HS-glycoprotein	6.05	6.12	1.01
8	Coagulation factor X	1.16	4.17	3.59
9	Apolipoprotein A-II	10.69	4.65	0.44
10	Hemoglobin subunit alpha	1.40	0.43	0.31
11	Protein AMBP	1.22	0.67	0.55
12	Apolipoprotein D	3.20	0.41	0.13
13	Hemoglobin fetal subunit beta	2.29	0.85	0.37
14	Apolipoprotein C-III	5.63	1.61	0.29
15	Aggrecan core protein	0.55	0.31	0.56
16	Hemoglobin subunit beta	1.31	0.63	0.48
17	Apolipoprotein E	1.22	0.10	0.08
18	Oxidoreductase-like domain-containing protein 1	5.29	7.90	1.49
19	Pinin	5.29	7.90	1.49

**Table 2:** Summary table presenting the proportions of individual proteins of the full and hard coronas, taking into account categorization by molecular weight.

Protein	0–37		37–50		50–75		75–100		150–250	
	FC	HC	FC	HC	FC	HC	FC	HC	FC	HC
Aggrecan core protein									0.31	0.55
Albumin					7.15	17.14				
Alpha-2-HS-glycoprotein			6.12	6.05						
Apolipoprotein A-I	2.80	16.39								
Apolipoprotein A-II	4.65	10.69								
Apolipoprotein C-III	1.61	5.63								
Apolipoprotein D	0.41	3.20								
Apolipoprotein E	0.10	1.22								
Coagulation factor X					4.17	1.16				
Complement C3									3.67	4.27
Hemoglobin fetal subunit beta	0.85	2.29								
Hemoglobin subunit alpha	0.43	1.40								
Hemoglobin subunit beta	0.63	1.31								
Inter-alpha-trypsin inhibitor heavy chain H3							2.82	0.93		
Oxidoreductase-like domain-containing protein 1	7.90	5.29								
Pinin							7.90	5.29		
Protein AMBP			0.67	1.22						
Prothrombin					35.37	15.62				
Vitamin K-dependent protein S							12.44	0.35		

## Data Availability

The data are available upon request from the corresponding authors.

## Supplementary Materials

The following supporting information can be downloaded at https://www.mdpi.com/article/10.3390/nano14231976/s1: Table S1: MLNC with hard corona; Table S2: MLNC with full corona; Table S3: MLNC with FBS and EMEM full corona.
